# High Prevalence of Multiple Antibiotic-Resistant, Extended-Spectrum β-Lactamase (ESBL)-Producing *Escherichia coli* in Fresh Seafood Sold in Retail Markets of Mumbai, India

**DOI:** 10.3390/vetsci7020046

**Published:** 2020-04-16

**Authors:** Asem Sanjit Singh, Binaya Bhusan Nayak, Sanath H. Kumar

**Affiliations:** Post Harvest Technology, ICAR-Central Institute of Fisheries Education (CIFE), Versova, Andheri (W), Mumbai 400061, India; sanjitasem21@gmail.com (A.S.S.); nayakbb@cife.edu.in (B.B.N.)

**Keywords:** MAR index, India, beta lactamase, MBL, disc diffusion, seafood, PCR

## Abstract

In this study, fresh seafood in retail markets was investigated for the antibiotic susceptibility patterns of the faecal indicator *Escherichia coli* and distribution of important β-lactamase encoding genes. *E. coli* were isolated from 50 (37 fish and 13 shellfish) fresh seafood samples and studied with respect to the phenotypic and genotypic characters of antibiotic resistance. Of 475 *E. coli* isolates from fresh seafood, 71.58% exhibited extended-spectrum β-lactamase (ESBL)-positive phenotypes. A high percentage of isolates were resistant to indicator cephalosporins cefotaxime (95%), cefpodoxime (90.88%) and ceftazidime (90.29%). Relatively higher susceptibilities were recorded against imipenem (74.41%), cefoxitin (66.76%) and meropenem (51.18%). The multiple antibiotic resistance (MAR) index of 97.35% of the isolates was above 0.18. The ESBL genes *bla*_CTX-M_, *bla*_SHV_ and *bla*_TEM_ were detected in 62.37%, 23.35% and 2.6% of *E. coli* isolates, respectively. The ESBL-producing isolates also harboured the metallo-β-lactamase-encoding genes *bla*_OXA_ (7.06%), *bla*_NDM_ (4.42%) and *bla*_VIM_ (0.88%). This study highlights the risk of dissemination of multidrug resistant *E. coli* in seafood consumer communities and also the need to improve the hygiene of the coastal waters, landing centres and the retail markets.

## 1. Introduction

*Escherichia coli* is a common inhabitant of the gastrointestinal tract of both humans and animals. Although *E. coli* is popular as an indicator of faecal contamination of water and foods, it is a significant food-borne human pathogen [1]. Contamination of seafood with *E. coli* as a consequence of contamination of coastal waters with domestic sewage is common in densely populated countries like India [2,3]. Such pathogenic *E. coli* can cause a range of infections, such as gastroenteritis, wound infections, septicaemia, urinary tract infections, etc. [4].

Resistance to multiple, clinically relevant antibiotics in *E. coli* has severely compromised the efficacy of antibiotic chemotherapy. Multidrug-resistant (MDR) *E. coli* is not restricted to clinical settings alone. Since humans and animals are sources of contamination of aquatic environments, MDR *E. coli* can be often found in seafood. Seafood as a vehicle of MDR *E. coli* is a serious concern, since such bacteria can be introduced via seafood to distant geographical regions, where such resistance patterns were unknown before [5]. Globally, beta-lactam antibiotics are among the most commonly prescribed drugs, which include penicillins, cephalosporins, cephamycins and carbapenems. In *E. coli*, as well as in several other members of the Enterobacteriaceae, production of one or more types of β-lactamases that hydrolyse β-lactam antibiotics is a key mechanism of resistance [6,7]. Among these, the extended-spectrum β-lactamases (ESBLs) can hydrolyse various types of β-lactam antibiotics, including third generation cephalosporins and monobactams, and confer resistance to bacteria against these antibiotics [7,8,9]. ESBLs have evolved from TEM-1, TEM-2 and SHV-1 β-lactamases by point mutation, and are generally susceptible to β-lactamase inhibitors (e.g., clavulanates and sulbactams) [10]. The second group of ESBLs, which are also derived from the classical TEM and SHV enzymes, are not susceptible to antibiotic-inhibitor combinations, except the piperacillin–tazobactam combination [10,11]. A relatively new category of ESBLs represented by CTX-M has a high affinity for cefotaxime and has been divided into five groups with more than 40 variants widely distributed throughout the world [12,13]. The CTX-M enzymes have independently evolved from chromosomally encoded β-lactamases of *Kluyvera* spp. and are susceptible to clavulanic acid [13].

Carbapenem-resistant Enterobacteriaceae (CRE) produce one or more carbapenemases which have varying abilities to hydrolyse carbapenems and differential susceptibilities to inhibition by clavulanic acid. These belong to the Ambler class A (KPC), class B lactamases, such as New Delhi metallo-β-lactamase (NDM), imipenemase (IMP), Verona integron-encoded metallo-β-lactamase (VIM) and the Class D oxacillinases (OXA-48/-181) [14,15]. CRE are resistant to most β-lactam antibiotics and often exhibit resistance to other antimicrobial classes, thus critically narrowing down the treatment options against them. Since these resistance markers are commonly located on mobile genetic elements, CRE are also important reservoirs of resistance genes that can be transmitted to clinical and environmental bacteria [16].

Seafood contamination of *E. coli* is primarily due to the faecal contamination of coastal waters. Post-harvest contamination of seafood with *E. coli* occurs in fish landing centres and markets. Past studies from India have reported the isolation of various pathogroups of *E. coli* from seafood [3,17,18,19] and also the presence of highly antibiotic resistant *E. coli*, such as the *bla*_NDM_-harbouring strains [20,21,22]. In the study reported here, we investigated the prevalence of antibiotic resistance in *Escherichia coli* isolated from seafood and the genetic factors responsible for resistance. The study highlights extensive occurrence of multidrug-resistant *E. coli* in seafood, which can be a potential health risk to seafood consumers and handlers as well.

## 2. Materials and Methods

### 2.1. Sample Collection, Isolation and Identification of Escherichia coli From Seafood

Fresh seafood samples (n = 50) comprising of fish (37) and shrimps (13) were collected from retail markets in north-western Mumbai, India, during October 2016 to February 2018. The samples were transported immediately to the laboratory in a chilled condition in sterile containers and processed for the isolation of *E. coli*. Equal portions from the head, belly and tail regions of finfish were pooled and 25 g of this pooled sample was used for analysis. Clams were opened using a sterile scalpel and the meat was collected from 2–3 specimens and pooled. Similarly, in the case of shrimps, the meat was collected and pooled. Twenty-five grams of the pooled meat was homogenized in 225 mL of tryptone broth (Hi-Media, Mumbai, India) in a stomacher (Seward Stomacher 80, Lab system, London, UK), serially diluted in tryptone water and spread plated on MacConkey agar (Hi-Media, Mumbai, India). Typical pink colonies, 3–5 from the useful dilution, were purified on Luria Bertani (LB) agar. Oxidase-negative isolates were subjected to biochemical tests for the identification of *E. coli* [23].

### 2.2. Antibiotic Susceptibility Tests

The production of extended-spectrum β-lactamase (ESBL) by *E. coli* isolates was detected by spotting on the chromogenic medium Hi-Chrome ESBL agar (Hi-Media, Mumbai, India), on which ESBL-positive isolates appear as blue/bluish-green colonies. Presumptive ESBL^+^ *E. coli* isolates were screened for resistance against the indicator cephalosporins cefotaxime (CTX; 30 µg), ceftazidime (CAZ; 30 µg) and cefpodoxime (CPD; 10 µg) by disk diffusion test. Resistant isolates were further tested against cefoxitin (CX; 30 µg), imipenem (IPM; 10 µg), meropenem (MRP; 10 µg), ertapenem (ETP; 10 µg), aztreonam (AT; 30 µg), amoxicillin/clavulanic acid (AMC; 30 µg), piperacillin/tazobactam (PIT; 100/10 µg) and ciprofloxacin (CIP; 5 µg). The zones of inhibitions were measured and interpreted as resistant or susceptible according to Clinical and Laboratory Standards Institute guidelines [24]. The multiple antibiotic resistance (MAR) index was determined as the ratio of the total antibiotics used to the number of antibiotics to which the bacterium was resistant [25].

### 2.3. Detection of ESBL and MBL Phenotypes

For phenotypic detection of ESBL production, the double disc synergy test was followed [26]. The test bacterium was grown in Mueller–Hinton (MH) broth to 0.5 McFarland units and a 0.1 mL of the broth culture was spread plated on MH agar. An amoxicillin/clavulanic acid (30/10 µg) disc was placed at the centre of the plate and ceftazidime (30 µg), cefotaxime (30 µg) and cefpodoxime (10 µg) discs were placed 20–30 mm away from the central disc. An extension in the zone of inhibition around the peripheral disc towards the centrally placed amoxicillin/clavulanic acid disc by at least 5 mm was considered as due to ESBL production. For further confirmation of an ESBL^+^ phenotype, the Triple ESBL detection Ezy MIC^TM^ Strip (Hi-Media, Mumbai, India) was used according to the manufacturer’s instructions. The upper half of the strip designated as “mixture^+^” is coated with ceftazidime, cefotaxime and cefepime plus clavulanic acid, while the lower half of the strip designated as “mixture” is coated with the same antibiotics in a reverse concentration gradient, but without an inhibitor. The strip was placed on the lawn culture of each test strain on MH agar and minimum inhibitory concentrations (MIC)s were read from the mixture and mixture^+^ ends of the strip. The isolate was considered ESBL-positive if the mix/mix^+^ value was ≥8. AmpC β-lactamase was inhibited by adding 200 µg/mL cloxacillin in the agar medium.

For detection of metallo-β-lactamase (MBL) production, the double disc diffusion synergy test was used [27]. The test bacterium was grown in Muller–Hinton (MH) broth as described above and spread onto MH agar plates. Two imipenem discs (10 μg), one of which contained 10 μL of 0.1M EDTA (292 μg), were placed 25 mm apart on the inoculated MH plates. MBL production was defined by a zone of inhibition of ≥ 4 mm around the imipenem–EDTA disc compared to the disc containing imipenem alone.

### 2.4. Detection of Antibiotic Resistance Genes

For detection of known antibiotic resistance genes, primers and PCR protocols previously described for *bla*_CTX-M_, [28], *bla*_SHV_ and *bla*_TEM_ [29], *bla*_NDM_ [30,31]_,_ *bla*_OXA_ [32] and *bla*_VIM_ [33] were used (Table S1). Bacterial DNA was extracted using the Wizard DNA kit (Promega, Madison, WI, USA) and PCR amplifications were done in a SimpliAmp thermal cycler (Thermo Fisher Scientific, Waltham, MA, USA).

## 3. Results and Discussion

Antibiotic-resistant bacteria are frequently encountered in raw seafood in India [20,34,35,36] and the incidence is alarmingly high in seafood harvested off the Mumbai coast [22,37]. The coastal city of Mumbai is densely populated, and the numerous creeks that lead to the sea act as conduits for draining untreated sewage. Every day, an estimated 2000 million litres of sewage is released into the Arabian sea. Direct contamination occurs from the population that live on the beaches without proper sanitation facilities. Retail markets too lack basic facilities to prevent contamination of fish and maintain hygiene. As a result, contamination of seafood with pathogenic and antibiotic-resistant bacteria is expected to occur right from the harvesting stage, till it reaches the consumer.

In this study, we investigated the occurrence of antibiotic-resistant *E. coli* in seafood, which directly represents the human cause of seafood contamination. The details of 50 seafood samples (37 fish and 13 shellfish) analysed for the presence of *E. coli* are shown in the Table 1. Overall, 896 bacterial colonies were isolated from MacConkey agar, of which 475 were identified as *E. coli*.

### 3.1. Distribution of ESBL^+^ E. coli in Seafood

Out of a total of 475 *E. coli* isolates, 340 (71.58%) were ESBL^+^ on the chromogenic medium (Table 1). Among 325 finfish isolates of *E. coli*, 261(80.30%) were ESBL^+^ and 79 (52.66%) out of 150 *E. coli* isolates from shellfish were also ESBL^+^. All the samples yielded at least one ESBL^+^ isolate, and all *E. coli* isolates from fish samples *Terapon jarbua, Otolithes cuvieri, Epinephelus diacanthus, Megalaspis cordyla* and *Anodontostoma chacunda* were ESBL^+^ (Table 1).

### 3.2. Antibiotic Resistance Profiles of E. coli

The antibiotic resistance patterns of 340 ESBL^+^ isolates are shown in Table 2. Studies reporting isolation of ESBL-producing *E. coli* from seafood in India are rare. We recently reported that 78.6% of the enterobacteria isolates from fresh seafood in Mumbai, India, were ESBL-positive [22]. ESBL^+^ Gram-negative bacteria were also isolated from effluents of a fish processing plant in Mangalore, India [38]. The latter study suggests that the contaminated fish can again be a source of environmental contamination with antibiotic-resistant bacteria, highlighting the need to treat effluents from fish processing plants. In Germany, ESBL- and AmpC-producing Enterobacteriaceae were detected in 21.3% of seafood samples, with *Klebsiella pneumoniae* and *E. coli* being the predominant species [39]. Sellera and colleagues [40] reported isolation of ESBL (CTX-M)-producing *E. coli* from wild fishes of a polluted area in the South Atlantic coast of Brazil. ESBL-producing *E. coli* of identical genotypes have been reported from fish, chicken and infected patients in Cambodia [41].

### 3.3. Molecular Characterization of ESBL^+^ E. coli

**Figure 1:**
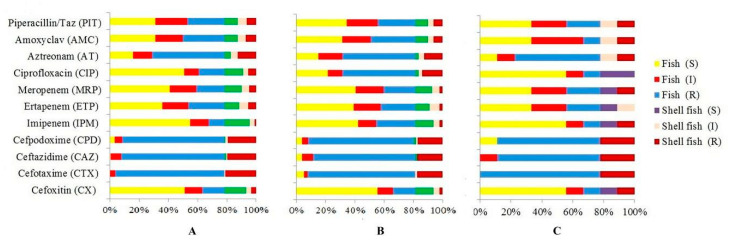
Antibiotic resistance profiles of *E. coli* harbouring *bla*_CTX-M_ (n = 212; fish 166, shellfish 46) (**A**), *bla*_SHV_ (n = 79; fish 64, shellfish 15) (**B**) and *bla*_TEM_ (n = 9; fish 7, shellfish 2) (**C**). R = resistant, I = Intermediate resistant and S = susceptible.

PCR investigation was carried out to identify the molecular mechanisms of antibiotic resistance in *E. coli* isolates of this study. The results are shown in Table 3. Among the ESBL genes tested in this study, *bla*_CTX-M_ was detected in the highest number of isolates (212, 62.35%). Sample-wise isolation of *bla*_CTX-M_^+^ *E. coli* is shown in Table 3. Finfish and shellfish yielded 166 (78.30%) and 46 (21.70%) of *bla*_CTX-M_^+^ isolates, respectively. The highest percentage of *bla*_CTX-M_^+^ *E. coli* was isolated from *Sardinella longiceps* (91.66%), followed by *Otolithes cuvieri* (89.47%), while the least percentage of such isolates was found in squid *Loligo duvauceli* (20%) (Table 3). The isolates exhibited highest resistance to cefotaxime (158 isolates, 74.53%). A high percentage of isolates (116 isolates, 69.88%) were susceptible to imipenem (Figure 1A). The isolates were largely susceptible to carbapenems and ciprofloxacin. The *bla*_SHV_ gene was detected in 79 (23.35%) isolates, 64 of which were from finfish and 15 from shellfish (Table 3). The isolates were commonly resistant to cefotaxime and ceftazidime (Figure 1B). Fifty-four (44%) isolates were susceptible to cefoxitin (Figure 1B). The *bla*_TEM_ gene was detected in the least number of isolates (9, 2.64%) (Table 3). The antibiotic susceptibility patterns of *bla*_TEM_-positive *E. coli* are shown in Figure 1C.

The CTX-M group of enzymes, which are commonly found in *E. coli*, confer resistance to cefotaxime, ceftriaxone and aztreonam [12,15]. Although CTX-M was originally found to be less active against ceftazidime, its variants, such CTX-M-15, hydrolyse ceftazidime efficiently [42,43,44]. In our study, 141 of 212 (66%) *bla*_CTX-M_-positive *E. coli* were susceptible to cefoxitin (Figure 1A). ESBL-producing *E. coli* is generally susceptible to cephamycins, thus making cephamycins, such as cefoxitin, viable options for treating ESBL-producing *E. coli* infections [45]. However, cefoxitin resistance due to loss of a porin has been reported in *K. pneumonia* and *E. coli* [46]. TEM, SHV and CTX-M-producing organisms have been reported from a variety of food-producing animals, such as poultry, swine, bovine, horse, rabbit, ostrich, wild boars, etc. [47].

In the present study, the *bla*_CTX-M_-harbouring *E. coli* were predominantly isolated compared to the *bla*_SHV_- or *bla*_TEM_-harbouring *E. coli*. The CTX-M group has the highest number of variants, with *E. coli* being the major producer of this enzyme [48]. The extraordinary mobility of plasmid and/transposon-located *bla*_CTX-M_ genes within clones of *E. coli* that are successful as pathogens has made CTX-M β-lactamase the most dominant ESBL currently [48]. The high prevalence of *E. coli* producing CTX-M β-lactamase in seafood might reflect on the local antibiotic consumption patterns as well as specific resistant clones circulating in the community.

### 3.4. Detection of Carbapenemase-Encoding Genes

**Figure 2:**
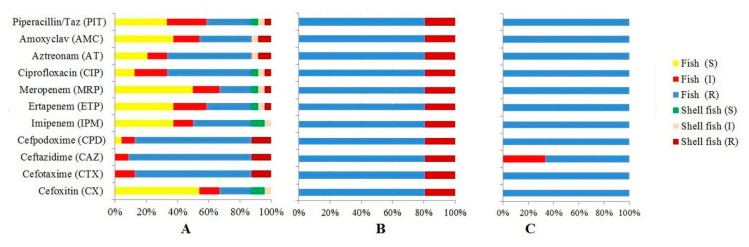
Antibiotic resistance profiles of *Escherichia coli* isolates harbouring *bla*_OXA_ (n = 24; fish 21, shellfish 3) (**A**), *bla*_NDM_ (n = 21, fish 17, shellfish 4 (**B**) and *bla*_VIM_ (n = 3, fish 3, shellfish 0) (**C**) genes. R = resistant, I = Intermediate resistant and S = susceptible.

Carbapenem-resistant Enterobacteriaceae (CRE) have become the biggest challenge in healthcare settings, as these bacteria are recalcitrant to most of the common antibiotics used. In our study, 21 (4.42%) isolates, 17 from fish and 4 from shellfish, harboured the *bla*_NDM_ gene (Table 3). The isolates were resistant to all tested antibiotics except colistin (Figure 2B). The present study strengthens our previous report of an alarmingly high occurrence of *bla*_NDM_-harbouring *E. coli* in fresh seafood [21,22].

The *bla*_OXA_ and *bla*_VIM_ genes were detected in 7.06% and 0.88% of the isolates, respectively. The antibiotic resistance profiles of these isolates are shown Figure 2. The *bla*_VIM_ was first reported in *Pseudomonas aeruginosa* [49]. Of the three isolates harbouring *bla*_VIM_, two isolates were resistant to all 11 antibiotics, while one had intermediate resistant to ceftazidime (Figure 2C). The VIM type metallo-β-lactamases are mostly reported from *Pseudomonas aeruginosa,* and from several non-related bacterial species like *Achromobacter xylosoxidans, E. coli, Klebsiella pneumoniae*, etc., which suggests its horizontal transfer ability among non-related species [50]. Worldwide, *bla*_VIM-2_ has been reported to be the dominant MBL variant [51]. Studies from India have reported VIM lactamase producing clinical isolates of *P. aeruginoasa* [52], *Acinetobacter baumannii* [53] and *E. coli* [54]. We did not find any reports of food-associated Gram-negative bacteria harbouring VIM and OXA type β-lactamase genes from India. Elsewhere, a recent study has reported the presence of *bla*_VIM_ in a food-associated *Vibrio parahaemolyticus* [55]. In our study, we screened *E. coli* isolates for ESBL production first using indicator cephalosporins, and this might have resulted in underestimation of certain ceftazidime-susceptible carbapenemase-producing *E. coli* (e.g., OXA-48) that lack ESBL and AmpC enzymes [56].

### 3.5. Multidrug Resistance in Seafood E. coli

Of the 340 isolates, 331 (97.35%) were resistant to two or more antibiotics with a MAR index of >0.18 (Table 4). A MAR index of more than 0.2 suggests contamination from high-risk sources where antibiotics are extensively used [25], and the majority of our isolates belonged to this category. All (100%) of the *E. coli* isolates recovered from nine samples were multidrug-resistant. These samples consisted of fish (*Sardinella longiceps*, *Otolithes cuvieri*, *Epinephelus diacanthus*, *Mene maculata, Priacanthus hamrur* and *Pampus argenteus*) and shellfish, such as crustaceans (*Acetes indicus* and *Metapenaeus dobsoni*) and clams (*Meretrix meretrix*). Twenty-one *E. coli* isolates were resistant to the entire range of 11 antibiotics tested.

In this study, we analysed 50 samples of fish and shellfish that belonged to 17 different species. Since we isolated multiple isolates of *E. coli* from a single sample, we selected one *E. coli* isolate from each species of fish or shellfish that was most resistant, 17 isolates in total. The antibiotic resistance profiles and the genotypes are given in the Table 5. Ten isolates were resistant to 11 antibiotics and susceptible to only colistin. Two isolates harboured all six β-lactamase genes tested. Other isolates showed high but varying levels of resistance to the tested antibiotics, and also harboured multiple β-lactamase genes.

## 4. Conclusions

The resistance patterns of *E. coli* observed in this study suggests anthropogenic sources of contamination of seafood. Release of raw or partially treated sewage into fishing areas is a major concern that requires the attention of the local regulatory bodies. Seafood can be a source of dissemination of antibiotic-resistant bacteria into the community. Incidence of MDR bacteria can also potentially jeopardise the international fish trade. In this regard, urgent measures are necessary to implement suitable policy to monitor the quality of coastal waters, ensure hygiene in retail fish markets and establish suitable testing methods to detect MDR bacteria in seafood.

## Figures and Tables

**Table 1:** Sample-wise distribution of *Escherichia coli and* ESBL^+^ isolates.

Sl. No.	Samples	No. Analysed	No. of *E. coli* Isolated	No. (%) ESBL^+^ *E. coli*
	Finfish			
1.	*Sardinella longiceps*	5	32	24 (75)
2.	*Terapon jarbua*	5	29	29 (100)
3.	*Otolithes cuvieri*	3	19	19 (100)
4.	*Epinephelus diacanthus*	2	15	15 (100)
5.	*Nemipterus randalli*	2	22	14 (64.28)
6.	*Mene maculata*	2	24	13 (53.84)
7.	*Coilia dussumieri*	3	45	32 (71.87)
8.	*Harpadon nehereus*	4	42	35 (82.85)
9.	*Trichiurus lepturus*	2	21	12 (58.33)
10.	*Priacanthus hamrur*	2	19	13 (69.23)
11.	*Megalaspis cordyla*	2	25	25 (100)
12.	*Anodontostoma chacunda*	3	13	13 (100)
13.	*Pampus argenteus*	2	19	17 (88.23)
	Shellfish			
14.	*Acetes indicus*	5	56	27 (48.21)
15.	*Metapenaeus dobsoni*	3	41	19 (73.68)
16.	*Meretrix meretrix*	3	26	18 (44.44)
17.	*Loligo duvauceli*	2	27	15 (26.66)
	Total	50	475	340 (71.58)

**Table 2:** Antibiotic susceptibility profiles of ESBL^+^ *E. coli* (n = 340) isolated in this study.

Antibiotic Tested	No. (%) Resistant	No. (%) Intermediate Resistant	No. (%) Susceptible
Cefoxitin (CX) 30 mcg	66 (19.41%)	47 (13.82%)	227 (66.76%)
Cefotaxime (CTX) 30 mcg	323 (95%)	12 (3.53%)	5 (1.47%)
Ceftazidime (CAZ) 30mcg	307 (90.29%)	26 (7.65%)	7 (2.05%)
Cefpodoxime (CPD) 10 mcg	309 (90.88%)	17 (5.0%)	14 (4.11%)
Imipenem (IPM) 10mcg	31 (9.11%)	56 (16.47%)	253 (74.41%)
Ertapenem (ETP) 10 mcg	107 (31.47%)	81 (23.82%)	152 (44.71%)
Meropenem (MRP) 10 mcg	87 (25.58%)	79 (23.24%)	174 (51.18%)
Ciprofloxacin (CIP) 5mcg	152 (44.71%)	43 (12.65%)	145 (42.65%)
Aztreonam (AT) 30 mcg	219 (64.41%)	57 (16.76%)	64 (18.82%)
Amoxyclav (AMC) 30 mcg	127 (37.35%)	83 (24.41%)	130 (38.24%)
Piperacillin/Taz 100/10mcg	114 (33.53%)	95 (27.94%)	131 (38.53%)

**Table 3:** Distribution of β-lactamase genes in *Escherichia coli* isolated in this study.

Samples Analysed (No.)	No. of *E. coli* Isolated	No. (%) ESBL^+^	No. (%) CTX-M^+^	No. (%) SHV^+^	No. (%) TEM^+^	No. (%) OXA^+^	No. (%) VIM^+^	No. (%) NDM^+^
*Sardinella longiceps (5)*	32	24 (75)	22 (91.66)	3 (12.5)	3 (12.5)	3 (12.5)	1 (3.13)	3 (9.38)
*Terapon jarbua (5)*	29	29 (100)	18 (62.06)	1 (3.45)	-	1 (3.45)	1 (3.45)	-
*Otolithes cuvieri (3)*	19	19 (100)	17 (89.47)	3 (15.79)	1(5.26)	1(5.26)	1 (5.26)	3 (15.78)
*Epinephelus diacanthus (2)*	15	15 (100)	13 (86.66)	-	1 (6.67)	1(6.67)	-	1 (6.66)
*Nemipterus randalli (2)*	22	14 (64.28)	5 (35.71)	5 (35.71)	-	-	-	2 (9.09)
*Mene maculata (2)*	24	13 (53.84)	5 (38.46)	2 (15.38)	-	-	-	-
*Coilia dussumieri (3)*	45	32 (71.87)	25 (78.13)	7 (21.86)	1 (3.13)	7 (21.88)	-	3 (6.66)
*Harpadon nehereus (4)*	42	35 (82.85)	27 (77.14)	7 (20)	1 (2.86)	4 (11.43)	-	1 (2.38)
*Trichiurus lepturus (2)*	21	12 (58.33)	5 (41.67)	9 (75)	-	-	-	2 (9.52)
*Priacanthus hamrur (2)*	19	13 (69.23)	3 (23.08)	7 (53.85)	-	-	-	-
*Megalaspis cordyla (2)*	25	25 (100)	9 (36)	11 (44)	-	1 (4)	-	2 (8)
*Anodontostoma chacunda (3)*	13	13 (100)	4 (30.77)	-	-	1 (7.69)	-	-
*Pampus argenteus (2)*	19	17 (88.23)	13 (76.47)	9 (52.94)	-	2 (11.76)	-	-
*Acetes indicus (5)*	27	27 (100)	23 (85.19)	12 (44.44)	-	1 (3.70)	-	2 (7.4)
*Metapenaeus dobsoni (3)*	26	19 (73.68)	11 (57.89)	2 (10.53)	2 (10.53)	1 (5.26)	-	-
*Meretrix meretrix (3)*	41	18 (44.44)	9 (50)	1 (5.56)	-	1 (5.56)	-	2 (4.88)
*Loligo duvauceli (2)*	56	15 (26.66)	3 (20)	-	-	-	-	-
**Total**	**475**	**340 (71.58)**	**212 (62.35)**	**79 (21.35)**	**9 (2.65)**	**24 (7.06)**	**3 (0.88)**	**21 (4.42)**

**Table 4:** Multiple antibiotic resistance (MAR) indices of *Escherichia coli*.

No. of Antibiotic	MAR Index	No. (%) of Isolates
1	0.09	9 (2.64)
2	0.18	7 (2.06)
3	0.27	71 (20.88)
4	0.36	11 (3.24)
5	0.45	102 (30)
6	0.55	13 (3.82)
7	0.64	53 (15.59)
9	0.82	41 (12.06)
11	1.00	21 (6.18)

**Table 5:** Characteristics of the *E. coli* isolates exhibiting the highest resistance phenotypes from each sample type.

Isolate No.	Source	Resistance Genotype	Antibiotic Resistance Profile	Antibiotic to which Susceptible
EC21	*Sardinella longiceps*	*bla*_CTX-M_, *bla*_SHV_, *bla*_TEM_, *bla*_NDM_, *bla*_OXA_, *bla*_VIM_	CX, CTX, CAZ, CPD, IPM, ETP, MRP, CIP, AT, AMC, PIT	CL
EC123	*Terapon jarbua*	*bla*_CTX-M_, *bla*_SHV_, *bla*_OXA,_*bla*_VIM_	CX, CTX, CAZ, CPD, MRP, AT	IPM, CIP, ETP, AMC, PIT
EC31	*Otolithes cuvieri*	*bla*_CTX-M_, *bla*_SHV_, *bla*_TEM_, *bla*_NDM_, *bla*_OXA_, *bla*_VIM_	CX, CTX, CAZ, CPD, IPM, ETP, MRP, CIP, AT, AMC, PIT	CL
EC91	*Epinephelus diacanthus*	*bla*_CTX-M_, *bla*_TEM_, *bla*_NDM_, *bla*_OXA_	CX, CTX, CAZ, CPD, IPM, ETP, MRP, CIP, AT, AMC, PIT	CL
EC81	*Nemipterus randalli*	*bla*_CTX-M_, *bla*_SHV_, *bla*_NDM_	CX, CTX, CAZ, CPD, IPM, ETP, MRP, CIP, AT, AMC, PIT	CL
EC13	*Mene maculata*	*bla*_CTX_, *bla*_SHV_	CX, CTX, CAZ, CPD, CIP, AT	IPM, MRP, ETP, AMC, PIT
EC221	*Coilia dussumieri*	*bla*_CTX-M_, *bla*_SHV_, *bla*_TEM_, *bla*_NDM_, *bla*_OXA_	CX, CTX, CAZ, CPD, IPM, ETP, MRP, CIP, AT, AMC, PIT	CL
EC201	*Harpadon nehereus*	*bla*_CTX-M_, *bla*_SHV_, *bla*_TEM_, *bla*_NDM_, *bla*_OXA_	CX, CTX, CAZ, CPD, IPM, ETP, MRP, CIP, AT, AMC, PIT	CL
EC303	*Trichiurus lepturus*	*bla*_CTX-M_, *bla*_SHV_, *bla*_NDM_	CX, CTX, CAZ, CPD, IPM, ETP, MRP, CIP, AT, AMC, PIT	CL
EC48	*Priacanthus hamrur*	*bla*_CTX_, *bla*_SHV_	CX, CTX, CAZ, CPD, CIP, AT, AMC, PIT	IPM, ETP, MRP
EC51	*Megalaspis cordyla*	*bla*_CTX-M_, *bla*_SHV_, *bla*_NDM_, *bla*_OXA_	CX, CTX, CAZ, CPD, IPM, ETP, MRP, CIP, AT, AMC, PIT	CL
EC253	*Anodontostoma chacunda*	*bla*_CTX_, *bla*_OXA_	CX, CTX, CAZ, CPD, CIP, AT, ETP	IPM, MRP, AMC, PIT
EC271	*Pampus argenteus*	*bla*_CTX-M_, *bla*_SHV_, *bla*_OXA_	CX, CTX, CAZ, CPD, CIP, AT, PIT	IPM, ETP, MRP, AMC
EC281	*Acetes indicus*	*bla*_CTX-M_, *bla*_SHV_, *bla*_NDM_, *bla*_OXA_	CX, CTX, CAZ, CPD, IPM, ETP, MRP, CIP, AT, AMC, PIT	CL
EC253	*Metapenaeus dobsoni*	*bla*_CTX-M_, *bla*_SHV_, *bla*_TEM_, *bla*_OXA_	CX, CTX, CAZ, CPD, IPM, CIP, AT, AMC, PIT	IPM, AMC, PIT
EC305	*Meretrix meretrix*	*bla*_CTX-M_, *bla*_SHV_, *bla*_NDM_, *bla*_OXA_	CX, CTX, CAZ, CPD, IPM, ETP, MRP, CIP, AT, AMC, PIT	CL
EC131	*Loligo duvauceli*	*bla*_CTX-M_	CX, CTX, CAZ, CPD, CIP	IPM, ETP, MRP, AMC, PIT

## Supplementary Materials

The following are available online at https://www.mdpi.com/2306-7381/7/2/46/s1, Table S1: Oligonucleotide primers used in this study for the detection of antibiotic resistance genes.
