# Supplementary material for: High Prevalence of Multiple Antibiotic-Resistant, Extended-Spectrum β-Lactamase (ESBL)-Producing Escherichia coli in Fresh Seafood Sold in Retail Markets of Mumbai, India
Source: Vet Sci. 2020 Apr 16;7(2):46. doi: 10.3390/vetsci7020046 (PMC7356741; doi:10.3390/vetsci7020046)
Supplement: Supplementary file 1 [file vetsci-07-00046-s001.pdf]

Table S1. Oligonucleotide primers used in this study for the detection of antibiotic resistance genes.

Sl. No.	Primer name	Target genes	Primer sequences (5′-3′)	Product size (bp)	References
1.	TEM-F<br>TEM-R	bla_{TEM}	AAAATTCTTGAAGACG<br>TTACCAATGCTTAATCA	1080	[29]
2.	SHV-F<br>SHV-R	bla_{SHV}	TTAACTCCCTGTTAGCCA<br>GATTTGCTGATTTCGCCC	786	[29]
3.	CTX-F<br>CTX-R	bla_{CTX}	CGATATCGTTGGTGCCATA<br>TTTGCGATGTGCAGTACCAGTAA	544	[28]
4.	NDM-F1<br>NDM-R1	bla_{NDM}	ATGGAATTGCCCAATATTATGCAC<br>TCA GCG CAG CTT GTC GGC′	815	[30]
5.	NDM-F2<br>NDM-R2	bla_{NDM}	GGG CAGTCG CTT CCAACGGT<br>GTA GTG CTC AGT GTCGCAT	475	[31]
6.	OXA -1A<br>OXA -1R	bla_{OXA}	CCAAAGACGTGGATG<br>GTTAAATTCGACCCCAAGTT′	540	[32]
7.	VIM-F<br>VIM-R	bla_{VIM}	GATGGTGTTTGGTCGCATA<br>CGAATGCGCAGCACCAG	390	[33]
